# Translational Read-Through Therapy of *RPGR* Nonsense Mutations

**DOI:** 10.3390/ijms21228418

**Published:** 2020-11-10

**Authors:** Christine Vössing, Marta Owczarek-Lipska, Kerstin Nagel-Wolfrum, Charlotte Reiff, Christoph Jüschke, John Neidhardt

**Affiliations:** 1 Human Genetics, Faculty of Medicine and Health Sciences, University of Oldenburg, 26129 Oldenburg, Germany; Christine.Voessing@uol.de (C.V.); Marta.Owczarek-Lipska@uol.de (M.O.-L.); christoph.jueschke@uol.de (C.J.); 2 Institute of Developmental Biology and Neurobiology, Johannes Gutenberg-University of Mainz, 55099 Mainz, Germany; nagelwol@uni-mainz.de; 3 Institute of Molecular Physiology, Johannes Gutenberg-University of Mainz, 55099 Mainz, Germany; 4 Eye Center, Albert-Ludwigs-University of Freiburg, 79106 Freiburg, Germany; cpolo@gmx.de; 5 Research Center Neurosensory Science, University of Oldenburg, 26129 Oldenburg, Germany; 6 Joint Research Training Group of the Faculty of Medicine and Health Sciences, University of Oldenburg, Germany and the University Medical Center Groningen, 9700 Groningen, The Netherlands

**Keywords:** RPGR, transcriptional therapy, read-through therapy, cilia, cilium, treatment, stop mutation, nonsense mutation, Ataluren, PTC124

## Abstract

X-chromosomal retinitis pigmentosa (RP) frequently is caused by mutations in the retinitis pigmentosa GTPase regulator (*RPGR*) gene. We evaluated the potential of PTC124 (Ataluren, Translama^TM^) treatment to promote ribosomal read-through of premature termination codons (PTC) in *RPGR*. Expression constructs in HEK293T cells showed that the efficacy of read-through reagents is higher for UGA than UAA PTCs. We identified the novel hemizygous nonsense mutation c.1154T > A, p.Leu385* (NM_000328.3) causing a UAA PTC in *RPGR* and generated patient-derived fibroblasts. Immunocytochemistry of serum-starved control fibroblasts showed the RPGR protein in a dot-like expression pattern along the primary cilium. In contrast, RPGR was no longer detectable at the primary cilium in patient-derived cells. Applying PTC124 restored RPGR at the cilium in approximately 8% of patient-derived cells. RT-PCR and Western blot assays verified the pathogenic mechanisms underlying the nonsense variant. Immunofluorescence stainings confirmed the successful PTC124 treatment. Our results showed for the first time that PTC124 induces read-through of PTCs in *RPGR* and restores the localization of the RPGR protein at the primary cilium in patient-derived cells. These results may provide a promising new treatment option for patients suffering from nonsense mutations in *RPGR* or other genetic diseases.

## 1. Introduction

Retinitis pigmentosa (RP, OMIM #268000) belongs to a group of genetically heterogeneous retinal disorders which are characterized by the progressive degeneration of photoreceptor cells, often leading to total blindness. RP can be caused by mutations in more than 60 genes (for reference see Retinal Information Network, Retnet, http://sph.uth.tmc.edu/Retnet/) and affects approximately one in 3000 to 4000 individuals worldwide [1,2]. RP can either occur as an isolated non-syndromic or as part of a complex syndromic phenotype. It may follow the inheritance pattern of autosomal-dominant, autosomal-recessive or X-linked traits [3].

X-linked RP (XLRP) is characterized by an early disease onset and a rapid progression of the visual defects [4]. Mutations in the *Retinitis Pigmentosa GTPase Regulator (RPGR)* gene are the most common causes of XLRP. Approximately 80% of families affected by XLRP and up to 15% of the simplex cases are caused by mutations in *RPGR* [5,6,7]. The human *RPGR* gene consists of 19 constitutive exons, as well as the alternatively spliced exon ‘open reading frame 15’ (ORF15). Among other RPGR isoforms, two widely expressed transcripts, *RPGR^ex1–19^* (full-length RPGR) and *RPGR^skip14/15^* (transcript that skips the constitutive exons 14 and 15 during splicing), and the retina-specific transcript *RPGR^ORF15^* have been described [8,9,10,11]. The RPGR proteins show a characteristic dot-like expression pattern along the transition zone and axoneme of primary cilia in cultured cells [12]. Comparably, the RPGR^ORF15^ protein localizes predominantly along the connecting cilium of photoreceptor cells [13,14,15]. All major isoforms share the N-terminal region including the tandem repeat structure which is homologous to the regulator of chromosome condensation (RCC1) protein, termed RCC1-like domain (RLD). The RLD was bioinformatically predicted to function as a guanine nucleotide exchange factor (GEF) for small GTPases, but so far GEF activities have not been associated with RPGR [16,17].

Disease-causing mutations in the *RPGR* gene were reported for exons 1 to 15 and ORF15 [18,19]. In contrast, pathogenetic sequence alterations were not observed in the 3´-exons 16 to 19 [20]. Although the mutational spectrum of the *RPGR* gene is diverse, up to 20% of the reported mutations in *RPGR* are nonsense mutations (for reference see Human Gene Mutation Database; http://www.hgmd.cf.ac.uk/ac/index.php), leading to premature termination codons (PTCs). PTC-carrying transcripts may be targeted for degradation by the nonsense mediated mRNA decay (NMD) pathway [21,22]. Furthermore, translation of PTC-containing mRNAs may result in truncated proteins and frequently are considered to lead to loss-of-function of the protein [21,23,24,25]. Several therapeutic approaches are under development or in clinical testing with the aim to treat hereditary retinal diseases [19,25,26,27,28,29]. Few approaches are already available to patients [30,31,32]. Therapies to specifically restore the function of a truncated RPGR protein caused by a nonsense mutation are not available to date, although translational-read-trough approaches have been described to suppress PTCs, thereby restoring the expression of full-length protein [25,29]. Aminoglycoside antibiotics such as gentamicin or geneticin (G-418) are used for translational read-through therapies in a variety of contexts and are known to suppress PTCs by the incorporation of a near-cognate amino acid at the site of the PTC [25,33,34]. Significant advances of read-through therapies using aminoglycosides have been described for different inherited diseases, where the therapeutic approach often results in partial but significant restoration of full-length protein expression, but may also cause ototoxicity, retinal toxicity, nephrotoxicity or other toxic side effects at the therapeutically relevant concentrations [35,36,37,38,39,40,41].

The translational read-through inducing reagent PTC124 (Ataluren; Translama^TM^, 3-[5-(2-fluorophenyl)-1,2,4]-oxadiazol-3-yl]benzoic acid) was described to have a promising biocompatibility in many human tissues with low cellular toxicity [42]. PTC124 has no structural similarity to aminoglycosides and the mechanism of action is not completely understood [42]. In vitro studies using patient-derived induced pluripotent stem cells and fibroblasts as well as in vivo studies using rodent models for RP showed, that PTC124 is able to promote translational read-trough of PTCs and a partial rescue of the full-length protein was observed [43,44,45,46].

To help patients suffering from disease-associated nonsense mutations in the *RPGR* gene, a treatment with the reagent PTC124 presents a promising therapeutic strategy. In this study, we evaluated the potential of a read-through therapy using PTC124 to promote translational read-through in a patient-derived fibroblast cell line that shows a hemizygous nonsense mutation in exon 10 of the *RPGR* gene (c.1154T > A, p.Leu385*).

## 2. Results

### 2.1. Analysis of the Mutation Spectrum in the RPGR Gene

**Figure 1:**
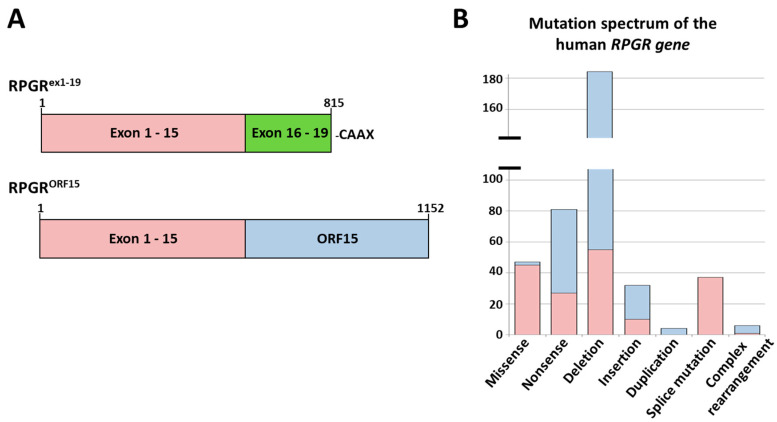
*RPGR* isoforms *RPGR^ex1–19^* and *RPGR^ORF15^* and the distribution of reported disease-causing *RPGR* mutations. (**A**) The transcript *RPGR^ex1–19^* is widely expressed, consists of exon 1 to 19 (exon 1–15 (red); exon 16–19 (green)) and encodes for a 815 amino acid protein containing an isoprenylation site (CAAX) at the C-terminus. Of note, the widely expressed isoform RPGR^skip14/15^, which skips exons 14 and 15 during splicing, is not shown. The retina-specific isoform *RPGR^ORF15^* includes exon 1 to 15 (red) and the exon ORF15, a highly repetitive purine rich domain (blue). (**B**) Reported *RPGR* mutations include 391 disease-causing sequence alterations. Deletions were most frequently identified, followed by nonsense mutations and missense mutations. Disease-causing mutations were not reported for exons 16 to 19.

We analysed the disease-causing mutations reported for the *RPGR* gene (Figure 1A,B) (for reference see: public version of the Human Gene Mutation Database (HGMD; http://www.hgmd.cf.ac.uk/ac/index.php)). In total, 391 disease-associated pathogenic variants (mutations) were reported in the *RPGR* gene affecting exon 1–15 and exon ORF15. So far, mutations were not reported for exons 16 to 19 (Figure 1B), although 175 mutations were reported in the region encoded by exon 1 to 15 in addition to 216 mutations identified in exon ORF15. The most frequently reported disease-associated alterations in the *RPGR* gene were deletions (184 deletions were published; 129 deletions were observed in ORF15 and 55 in exon 1 to 15).

Interestingly, nonsense mutations caused by single base substitutions, were the second most common mutation type. Approximately 20% of all reported mutations in the *RPGR* gene resulted in a PTC. So far, 81 nonsense mutations were observed in *RPGR* (Figure 1B), with a diverse distribution over the constitutive exons 1 to 15 and ORF15. Exons 1 and 12 did not show nonsense mutations.

### 2.2. A New Nonsense Mutation in Exon 10 Leading to a PTC

Surprisingly, the analyses of the *RPGR* mutation spectrum revealed that nonsense mutations in exons 1 through 15 were frequently identified in exon 10. Exon 10 was associated with 7 PTC-causing mutations, whereas additional exons between 1 and 15 only showed 1, 2 or 3 nonsense mutations per exon (with a mean of 1.67 nonsense mutations per exon).

**Figure 2:**
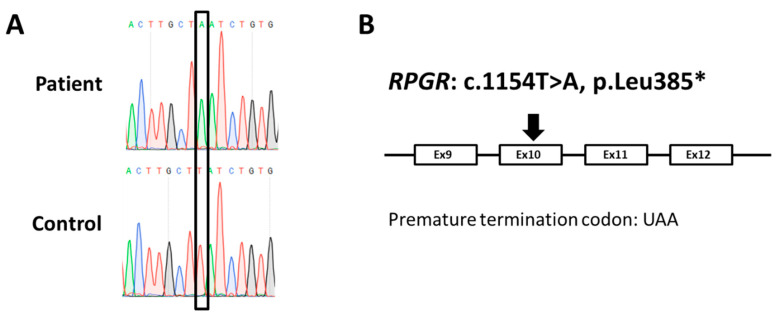
A novel nonsense mutation identified in the *RPGR* gene. (**A**) Electropherograms of the index patient and a control. Sanger sequencing of the index patient´s genomic DNA showed a hemizygous adenine sequence alteration at position c.1154 in exon 10 of the *RPGR* gene. In contrast, the unaffected control (male) contained a thymine base at the same position, representing the reference sequence of *RPGR*. The black frame marks the sequence alteration at nucleotide c.1154 in *RPGR* (*RPGR* reference sequence NM_000328.3). (**B**) Schematic drawing of the *RPGR* gene illustrating the position of the mutation. We identified a single base substitute (c.1154T > A) in exon 10 of *RPGR* leading to the premature termination codon UAA (p.Leu385*).

Herein, we identified an additional and novel disease-associated mutation in exon 10 of *RPGR* (Figure 2A,B). The disease-associated mutation was the nonsense mutation c.1154T > A, p.Leu385* (*RPGR* reference sequence NM_000328.3) affecting all major isoforms of *RPGR*. It was predicted to lead to truncated RPGR^ex1–19^, RPGR^skip14/15^, and RPGR^ORF15^ proteins. Consequently, the mutation c.1154T > A in the *RPGR* gene likely causes a loss of function of the RPGR protein.

### 2.3. The Read-Through Inducing Reagents Partially Restored RPGR Expression

**Figure 3:**
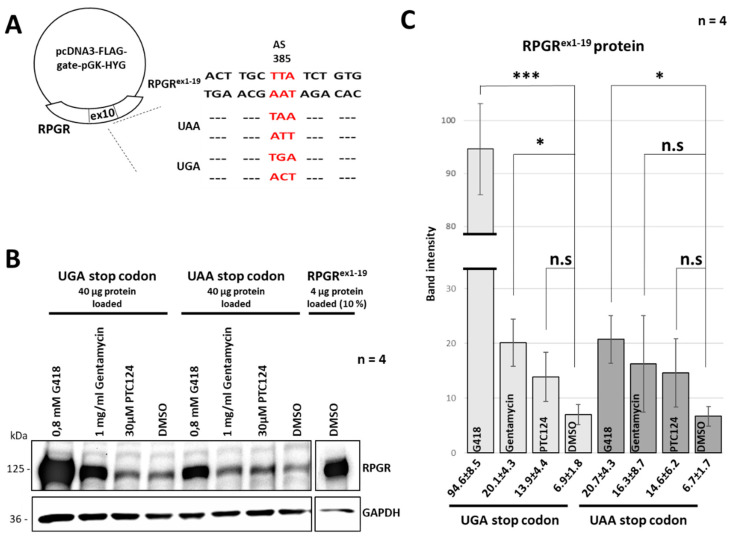
PTC124 treatment partially restores RPGR expression. (**A**) RPGR expression constructs encoding the full-length RPGR protein encoded by exon 1 through 19. Expression constructs were mutated with either an UAA stop codon (RPGR- UAA stop codon) or a UGA stop codon (RPGR- UGA stop codon) at amino acid position 384. The RPGR expression construct encoding the reference RPGR^ex1–19^ protein was used as a control. Sequence alterations in the expression construct are highlighted in red. (**B**) *RPGR* expression constructs were transfected into HEK293T cells and treated with either a single dose of PTC124, G418, Gentamycin or DMSO for 48 h. Full-length RPGR was detected applying a polyclonal RPGR antibody directed against amino acids 379 to 509 of RPGR. The treatments with G418 and gentamycin partially restored the full-length RPGR protein expression. Read-through efficacies of the reagents PTC124, G418 and gentamycin were compared to samples incubated with DMSO. The experiments were replicated four times (n = 4). GAPDH (36 kDa) was used as a loading control. Protein sizes are indicated on the left side in kDa. (**C**) Quantification of the band intensities suggested that all read-through-inducing reagents led to an upregulation of the expression of full-length RPGR protein. Levels were normalized to GAPDH signal intensities and compared to the negative control (DMSO). The quantification was based on four independent replicates (n = 4). Error bars represent standard deviation. Asterisks: significant differences; n.s: non-significant, *: *p* ≤ 0.05, ***: *p* ≤ 0.001.

We asked the question whether treatments with read-through-reagents are efficient to overcome the deleterious effects of *RPGR* nonsense mutations. Efficacies of translational read-through therapies depend on the PTC type and the surrounding nucleotides [42]. Consequently, we analyzed the effect of different read-through inducing reagents on an UAA stop codon (considered to show the lowest read-through efficacy) and compared it to an UGA stop codon (considered to show the highest read-through efficacy) [33,42]. We generated expression constructs encoding the full-length RPGR protein from exon 1 to 19. These constructs were engineered either with an UAA or with an UGA stop codon. The mutations were introduced at nucleotide c.1154 resulting in a PTC at amino acid position 385. The position of the mutation was identical to the nonsense mutation of the patient described herein (Figure 3A). The RPGR expression construct encoding the reference RPGR^ex1–19^ protein was used as control. Expression constructs were transfected into HEK293T cells and treated either with a single dose of PTC124 (30 µM), G418 (0.8 mM), gentamicin (1 mg/mL) or DMSO (negative control) for 48 h. We performed Western blot analyses using a polyclonal anti-RPGR antibody to compare the efficacies of the read-through inducing reagents (Figure 3B). Signal intensities of the full-length RPGR protein in Western blot analyses indicated that the treatment efficacies with PTC124, G418 and Gentamycin were different. The negative control DMSO showed weak signals either detecting the endogenous expression of RPGR in HEK293T cells or basal read-through, as previously detected in transfected HEK293T cells [41]. Results were verified in four independent experiments. We quantified the RPGR-specific signals comparing the reagents PTC124, G418 and gentamycin to the negative control DMSO (Figure 3C). The relative band intensities of the RPGR protein were normalized to the relative band intensities of the corresponding GAPDH loading control. All read-through inducing reagents either showed a significant effect or a promising tendency to upregulate the expression of full-length RPGR. The read-through efficacy detected in the construct carrying the UAA stop codon was less efficient compared to the one carrying the UGA stop codon. Treatment with G418 led to the highest increase of RPGR expression, with a clearly stronger efficiency on UGA than UAA stop codons. These findings showed that the treatment with read-through reagents G418 and Gentamycin was able to increase the expression of the full-length RPGR protein. A treatment of the UGA stop codon with PTC124 suggested a trend towards increasing expression levels of full length RPGR.

### 2.4. Nonsense-Mediated mRNA Decay Acts on the Truncated RPGR Transcript

**Figure 4:**
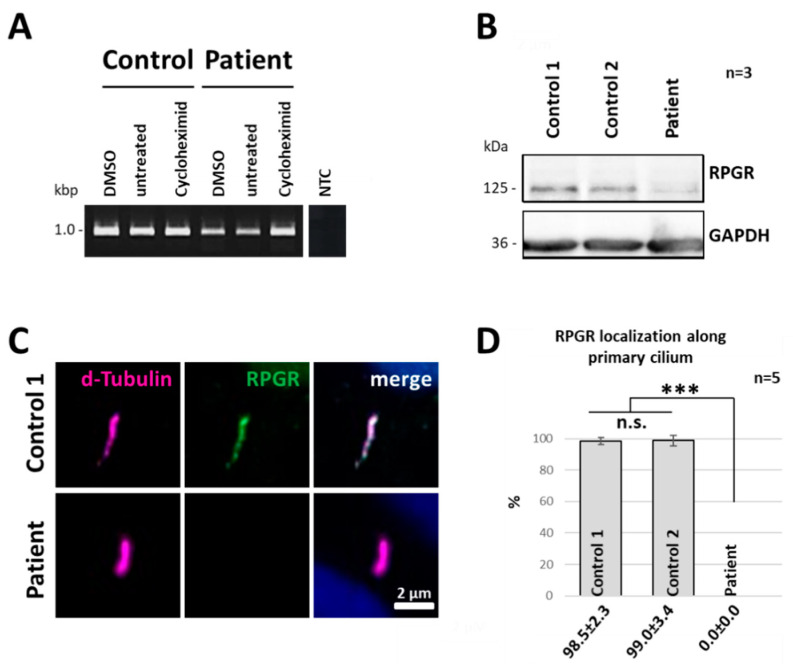
Patient-derived dermal fibroblasts lack RPGR at primary cilia. (**A**) Influence of NMD on *RPGR* transcript levels in patient-derived and control fibroblasts. Cells were treated with 100 µg/mL cycloheximide (CHX) for 4 h. DMSO-treated and untreated cells were used as controls. RT-PCR analyses included exon 9 to 11 of the RPGR transcript and were performed using equal amounts of RNA in each sample. NTC: non-template control. (**B**) Western blot analyses showed a strongly reduced level of RPGR protein expression (125 kDa) in the patient-derived cell line. Residual signals in the patient might correspond to basal spontaneous read-through. In contrast, RPGR was detected in two unaffected control cell lines. GAPDH (36 kDa) was used as a loading control. Molecular weight is indicated in kDa. (**C**) Immunocytochemistry (ICC) of RPGR at primary cilia. Control cells showed a dot-like expression pattern of RPGR (green) along the axoneme of the cilium (magenta). In the patient-derived fibroblasts, RPGR expression was not detected. D-tubulin was used as a marker for the primary cilium. (**D**) Quantification of cilia showing RPGR along the ciliary axoneme. A highly significant reduction of RPGR signals at primary cilia was detected in the patient-derived cell line. More than 90% of the control cells showed RPGR at the primary cilium, whereas RPGR was not detected along the cilium in the patient cell line (among 250 primary cilia, none showed RPGR staining). Experiments were independently repeated 5 times (n = 5). Error bars represent standard deviation. Asterisks: significant differences; n.s: non-significant, ***: *p* ≤ 0.001.

We used patient-derived skin fibroblasts as a human cellular model system to study the effects of nonsense mutations in the *RPGR* gene. Nonsense mutations or PTCs frequently promote the degradation of transcripts by NMD. To analyse whether the novel nonsense mutation described herein led to the degradation of the mutated *RPGR* transcript, we compared mRNA levels of *RPGR* in patient-derived dermal fibroblasts from the index patient and two unaffected controls. In order to block the NMD pathway, we applied the NMD inhibitor cycloheximide (CHX) (Figure 4A). The analyses of the CHX treatments were performed under highly comparable conditions using the same RNA concentrations in all samples. Results were compared to DMSO-treated (DMSO was used as detergent to dissolve CHX) and untreated cells. In control cells, we did not observe differences in *RPGR* expression between CHX and untreated samples, indicating that the NMD pathway is not acting on the *RPGR* reference transcripts. In contrast, RT-PCR analyses of the DMSO and untreated cells of the index patient showed weaker *RPGR* expression levels compared to control cells, indicating a reduced concentration of the *RPGR* transcript. Furthermore, CHX-treatment of the index patient-derived fibroblasts resulted in an increase of the RT-PCR band intensity, at a level comparable to the expression level of the controls (Figure 4A and Supplementary Figure S2). Thus, our results suggested that the novel nonsense mutation targeted the *RPGR* mRNA transcript to the NMD pathway in patient-derived fibroblasts.

### 2.5. RPGR Protein Expression Is Reduced in Patient-Derived Cells

Western blot analyses were applied to verify the consequences of PTC-containing transcripts on protein expression of RPGR. Using a polyclonal RPGR antibody raised against the peptide sequence from exon 10 to 13, we were able to detect the full-length protein RPGR^ex1–19^ (125 kDa) in unaffected control fibroblasts (Figure 4B). In comparison, only a faint band at the size of 125 kDa was detected in patient-derived cells, suggesting that the PTC in the *RPGR* gene led to reduced protein expression of full-length RPGR (Figure 4B). The faint band at 125 kDa may resulted from a spontaneous read-through of the p.Leu385*X nonsense mutation, similar as described before [41]. Nevertheless, our data indicated that the nonsense mutation in *RPGR* exon 10 caused a downregulation of the RPGR protein. Full-length RPGR expression was clearly reduced in the patient-derived cell line.

### 2.6. RPGR Protein Is Not Detectable at the Primary Cilium of Patient-Derived Cells

We previously demonstrated that RPGR localizes to primary cilia of fibroblasts [12]. To analyse whether the nonsense mutation c.1154T > A in the index patient influenced the subcellular localization of the RPGR protein, we starved healthy control fibroblasts and the patient-derived fibroblasts for 48h to trigger ciliogenesis and subsequently performed immunofluorescence staining of RPGR at the primary cilium. The axoneme of the primary cilium was labelled by a detyrosinated α-tubulin (d-tubulin) antibody. The RPGR protein was detected by a polyclonal RPGR antibody that was raised against the amino acid sequences encoded by exon 10 to 13 (amino acids 379 to 509; reference sequence NP_000319). In control fibroblasts, we previously found RPGR in a dot-like expression pattern along the axoneme with a preferred localization to the presumed transition zone of primary cilia [12]. We confirmed these results in unaffected controls and found dot-like expression patterns of RPGR along the ciliary axoneme (Figure 4C). The RPGR signal intensity decreased within the axoneme. In contrast, in the index patient-derived dermal fibroblasts RPGR-specific expression patterns along the ciliary axoneme or at the presumed transition zone of the primary cilium were not detectable. These results were verified in five independent experiments, in total analysing 250 cilia in each, the index patient and control cell lines (Figure 4D). These findings indicated that the nonsense mutation described herein caused a strongly reduced protein expression, and/or protein degradation, and/or inability of the truncated protein to enter the primary cilium.

### 2.7. PTC124 Partially Restores Protein Expression and Localization of RPGR in Patient-Derived Fibroblasts

**Figure 5:**
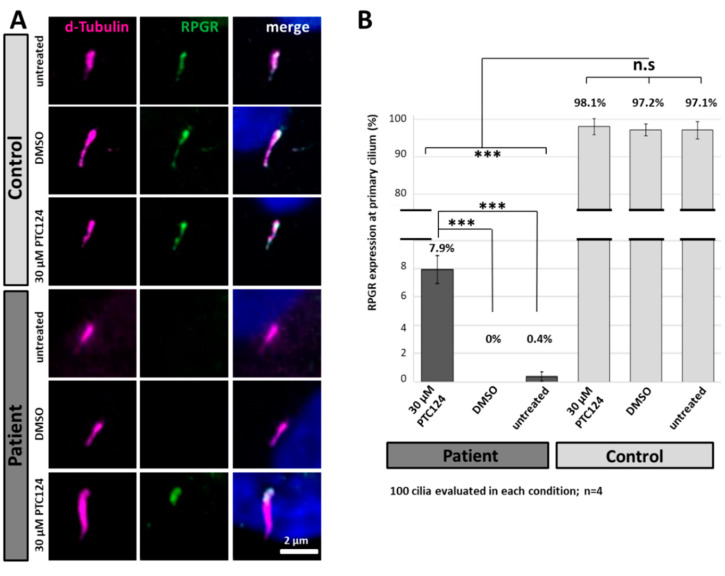
PTC124 treatment partially restores RPGR protein expression and localization in patient-derived fibroblasts. (**A**) Immunocytochemistry of the primary cilium (d-tubulin, magenta) and the subcellular localization of RPGR (green). Read-through therapy was applied using a single dose of 30 µM PTC124. Control cell lines showed similar RPGR expression patterns in each condition. Neither DMSO nor PTC124 did influence the ciliary localisation of RPGR in control cells. Untreated and DMSO treated fibroblasts derived from the patient showed no RPGR at the primary cilium. PTC124 treatment restored RPGR localization at the primary cilium, predominantly detected at one end of the axoneme. Scale bar: 2 µm. (**B**) Quantification of RPGR signals at the primary cilium in the index patient confirmed differences between PTC124-treated and control conditions. Approximately 8% of the PTC124-treated patient-derived cells showed RPGR at the primary cilium. The PTC124 treatment showed no detectable influence in RPGR´s localization in the control cell lines. The analyses were reproduced in four independent replicates (n = 4). Error bars represent standard deviation. Asterisks: significant differences; n.s: non-significant, ***: *p* ≤ 0.001.

We analysed the ability of PTC124 (Ataluren) to induce translational read-through at the novel nonsense RPGR mutation p.Leu385* using the above characterized patient-derived fibroblasts (Figure 5A). Based on the clearly different subcellular localization of RPGR between patient-derived and control cells, we quantified the ciliary localization of RPGR after the PTC124 treatment. Patient-derived and control fibroblasts were treated for 48 h either with a single dose of 30 µM PTC124 or DMSO (negative control). In PTC124-treated patient-derived fibroblasts, immunofluorescence analysis showed a clear RPGR signal predominantly at the presumed transition zone of the primary cilium. In contrast, RPGR expression at the primary cilium was neither detected in DMSO treated nor in untreated cells of the patient. The control cell lines showed unaltered ciliary staining of RPGR at the primary cilium following PTC124 or DMSO staining (Figure 5A,B).

More specifically, a total of 400 cilia per cell line were analysed in four independent experiments (Figure 5B). More than 90% of the primary cilia in control cells contained RPGR. A PTC124-mediated read-through therapy in the index patients’ fibroblasts resulted in a significantly increased number of primary cilia showing RPGR-specific signals (7.9%). RPGR signals were almost undetectable in untreated and DMSO-treated patient-derived cells (0% and 0.4%, respectively). In summary, our results demonstrate that PTC124 (Ataluren) treatment partially restored the expression and localization of RPGR at the cilium.

## 3. Discussion

We provide a proof of concept study to evaluate the feasibility of read-through therapies to treat disease-causing nonsense mutations in *RPGR*. Patient-derived dermal primary cells with a hemizygous single base substitution in exon 10 of *RPGR* were used as a human model system to study the efficacies of the treatment. On the molecular level, our studies showed a successful therapeutic effect of read-through reagents to partially restore RPGR expression in heterologous expression systems and in patient-derived material. We further found indications that the transcript carrying the nonsense mutation is a target for degradation by the NMD pathway.

In patient-derived cells, the truncated RPGR protein was not detectable at the primary cilium. It seems likely that RPGR is either not transcribed due to NMD mechanisms, not stably expressed and/or unable to enter the cilium. A consequence of all nonsense mutations in *RPGR* is the removal of the isoprenylation site at the C-terminal end of the constitutive protein isoform, thus leading to a mis-localization of RPGR. Zhang and colleagues reported in 2019 that GFP-tagged expression constructs of the retina-specific isoform RPGR^ORF15^ and a construct encoding the exons 1 through 15, both lacking the isoprenylation site, did not localize to primary cilia [47]. In contrast, the full-length RPGR^ex1–19^ protein is able to locate to the primary cilium in cultured cell lines [47,48]. The isoprenylation site of cysteine 812, a posttranslational lipid modification at the C-terminal end of RPGR, seems essential to allow the RPGR proteins to enter the primary cilium.

We demonstrated that the read-through reagent PTC124 restores ciliary localisation of the human RPGR protein in patient-derived fibroblasts. We found a treatment efficacy of approximately 8%. The recovered RPGR localisation demonstrated that the PTC was partially suppressed and suggested that a continuous translation until the natural stop codon in exon 19 of *RPGR* was induced by PTC124. It seems likely that also the restored RPGR protein carried an isoprenylation at cysteine 812 which enabled the targeting of the RPGR protein to the primary cilium. While RPGR was frequently found along the primary cilium in controls (also compare to [12]), the PTC124-treated patient-derived fibroblasts rarely showed RPGR along the axoneme. We speculate that the rather low efficacy of the read-through treatment was insufficient to generate enough RPGR protein to detect both, the strong signal at the presumed transition zone and the weaker dot-like signal along the axoneme. However, we cannot exclude that the treatment of the mutated RPGR caused the protein to be retained at the presumed transition zone or that the treatment inhibited the intraflagellar transport (IFT). It has been reported that during PTC read-through of an UAA stop codon the three amino acids glutamine, lysine or tyrosine may be incorporated with a high frequency [49]. It seems likely that during PTC124-mediated read-through, instead of the original reference amino acid leucine, a near-cognate tRNA was incorporated at the PTC. This has the potential to result in an altered protein structure of the RCC1-like domain. Nevertheless, the treated RPGR was still able to be transported to the cilium, enter the cilium, and locate to the proper subcellular compartment of the presumed transition zone. Hence, most likely the functional properties of RPGR have been preserved during PTC read-through. Additional analyses of the RPGR function will be required to verify this conclusion, but, to the best of our knowledge, cell-based assays that reliably analyze RPGR-related functional properties have not yet been established.

It has been observed that PTC124 promotes translational read-through of all three stop codons with the highest efficiency for UGA stop codons followed by UAG and UAA. Furthermore, the efficiency of PTC124 is also influenced by the nucleotide context around the PTC and it was reported that the efficiency increases when a pyrimidine base, especially a cytosine, is following the nonsense codon [33,42]. The UAA stop codon of the patient described herein is followed by the pyrimidine nucleotide thymine, but still can be expected to be among the least sensitive PTCs to treat by read-through therapies. In the present study, we were able to restore ciliary localization of RPGR in approximately 8% of patient-derived cells. A UGA or UAG stop codon or a different nucleotide context surrounding the PTC might even have resulted in higher treatment efficiencies.

The application of read-through reagents as therapies to treat RPGR-related retinal degenerations and other symptoms might be of clinical relevance. Of note, the most common type of PTCs found in exon ORF15 is UAG (48%) followed by UAA (33%) and UGA (19%) (HGMD; http://www.hgmd.cf.ac.uk/ac/index.php). Furthermore, the high GC content of ORF15 increases the probability of a neighbouring cytosine base to the PTC. Thus, PTCs in the exon ORF15 might be interesting targets for translational read-through therapies using PTC124 or similar. To verify the effect of a PTC124-mediated read-through therapy on the progressive photoreceptor cell death, a rodent model of XLRP resembling a human nonsense mutation in the *RPGR* gene would be useful to confirm whether the treatment can ameliorate or even stop the *RPGR*-associated retinal degenerative processes.

The present study is the first to show that a read-through therapy can correct the pathogenic processes caused by a nonsense mutation in the *RPGR* gene. Documenting the potential of therapeutic agents like PTC124, the successful application of read-through therapies to treat PTCs in other genes and disorders were published in several previous studies [33,42,43,44,45,50]. Nevertheless, to rescue the disease-causing phenotype of XLRP caused by the nonsense mutation (p.R120X) in the *RP2* gene, translational read-through therapies were performed in two independent studies with controversial results. It has been reported that a read-through therapy in lymphoblastoid cells using the aminoglycoside gentamicin did not restore detectable levels of the full-length RP2 protein [51]. Interestingly, in patient-derived fibroblasts and iPSC-derived RPE cells carrying the same nonsense mutation in *RP2*, the treatment with PTC124 and the aminoglycoside G418 was successful, promoted translational read-through in both cell lines, and restore the RP2 protein with a treatment efficiency of 13 to 20% [44]. Although aminoglycosides such as G418 or gentamycin are known to promote translational read-through of all three stop codons with treatment efficiencies in a variety of contexts [34], it seems that cell type-specific factors influence the outcome of the treatment. In line with this hypothesis, other studies have also seen context dependent effects of the treatments: A read-through therapy using the aminoglycoside gentamycin in a rodent model for autosomal dominant RP (p.S334* in rhodopsin, transgenic rats) documented a significant amelioration of the retinal degeneration [45]. The same study found contrasting therapeutic efficacies for gentamycin in a second in vivo murine model affected by PTCs in *Rpe65*. A read-through effect was not detected, neither in the mutant *Rpe65* expression construct transfected in COS-7 cells, nor in mice. Furthermore, it cannot be excluded that patient-derived cell lines show divergent molecular mechanism to process stop mutations compared to in vivo disease models or to the patients´ retinal cells, implying that iPSCs and retinal organoids may be interesting tools to closely resemble retinal processes of patients [52]. The above mentioned data suggested caution in generalizing the therapeutic potential of read-through reagents to treat PTCs [45].

Furthermore, several clinical disadvantages of the aminoglycoside gentamycin were observed, e.g., the need to apply the agents by intramuscular or intravenous delivery or, even more significantly, the high level of nephron, retinal- and ototoxicity at therapeutic concentrations [53]. In contrast, the read-through reagent PTC124 has several clinical advantages. A well-tolerated biocompatibility for many human tissues with little toxic side effects has been described, especially for the retina [25,41]. Furthermore, PTC124 can be taken orally in a non-invasive administration. Currently, PTC124, under the commercial name Translarna^TM^ has been authorized for the treatment of Duchenne muscular dystrophy (DMD) and cystic fibrosis (CF) caused by in-frame nonsense mutations in the US, and has orphan drug status and conditional authorization for DMD in Europe [25]. 

Since XLRP caused by mutations in the *RPGR* gene is among the most frequent genetic disorders of the retina and shows an early age of onset, treatment options are currently under development. A promising therapeutic strategy for RPGR-related RP is a gene replacement therapy where adeno-associated virus (AAV) are injected subretinally to replace the isoform *RPGR^ORF15^* of the defective *RPGR* gene. Currently, three clinical trials are ongoing (NCT03116113, Nightstar Therapeutics (now Biogen Inc.) (https://www.biogen.com/en_us/home.html); NTC03316560, Applied Genetic Technologies Corporation (https://agtc.com/); NCT03252847, Meira GTx (https://meiragtx.com/)).

To complement the current attempts to develop therapeutic approaches to treat RPGR mutations, our proof-of-concept study demonstrated that PTC124 is able to promote translational read-through of PTCs in the *RPGR* gene restoring a significant amount of the mutated RPGR protein. Of note, 20% of all reported disease-causing alternations in the *RPGR* gene are nonsense mutations, a finding that provides an opportunity for read-through drugs to help an increasing number of patients suffering from *RPGR*-associated nonsense mutations. As a general conclusion, read-through therapies might present promising novel treatment options for RP or other genetic diseases.

## 4. Materials and Methods

### 4.1. Patients and Mutation Screening

The study was conducted according to the Declaration of Helsinki protocol and was approved by the local ethics committee (Medical Ethical Committee of the Univ. of Oldenburg, #2018-097; MHH ethical committee, #2576-2015). The consequences of the study were explained to the patients and unaffected controls. Written informed consent for clinical diagnostic testing and research applications was obtained from all participants before collecting blood samples and skin biopsies. Genomic DNA was extracted from EDTA blood samples as previously described [54] and used for molecular genetic analysis. To verify the mutation in exon 10 of *RPGR,* flanking intronic sequences and exon 10 were amplified by PCR using HotFire Tag Polymerase (Solis Biodyne, Tartu, Estonia) and analyzed by Sanger sequencing. Sequencing profiles of each family member were compared to the *RPGR* reference sequence NM_000328.3 (Snap Gene software, GSL Biotech LLC, San Diego, CA, USA).

### 4.2. Cell Culture of Fibroblast from Skin Biopsies

Skin biopsies were obtained from the index patient and two unaffected controls. The skin biopsies were cut into smaller pieces and transferred to a sterile culture flask. After the explants were attached to the bottom of the flask by air drying, minimal essential medium (MEM, Biowest, Nuaille, France) supplemented with 1.3% L-glutamine, 0.8% antibiotic and 20% fetal bovine serum (FBS) was added and incubated at 37 °C and 5% CO_2_. Fibroblasts growing out from the explants were harvested and transferred to 75 cm^2^ flaks for propagation and analyses. Cells were passaged when 80–90% confluency was reached. To inhibit NMD, cells were incubated with 100 µg/mL cycloheximide (Sigma-Aldrich, St. Louis, MO, USA) for 4 h at 37 °C and 5% CO_2_. For immunostaining 0.1 × 10^6^ fibroblast cells were seeded in 12-well plates and cultured for 24 h in normal MEM medium or in MEM containing 30 µM PTC124. Cilia generation was induced by starving the cells for 48h in FBS-free MEM with or without PTC124.

### 4.3. Plasmid and Site-Directed Mutagenesis

To generate expression constructs carrying different PTCs in the *RPGR* gene, PCRs were performed to amplify the human *RPGR* gene using Phusion Hot Start II DNA Polymerase (Thermo Fisher Scientific, Waltham, MA, USA). By site-directed mutagenesis (SDM), specific alterations in the RPGR sequence were generated. Primers used for SDM are listed in Supplementary Figure S1. By Gateway cloning (Gateway BP clonase II Enzyme mix; Invitrogen, Carlsbad, CA, USA) the generated SDM products, flanked by attB sites, were recombined into the donor vector (pDONR221) according to the manufacturer’s instruction. The Gateway LR Clonase Enzyme mix (Invitrogen, Carlsbad, CA, USA) was used to generate Destination clones (pcDNA3.1–3×FLAG-V5-ccdB; #87064, Addgene) according to the manufacturer’s instruction.

### 4.4. Cell Culture of HEK293T Cells and Transfection

Human embryonic kidney 293T cells (HEK293T) were cultured in Dulbecco’s modified Eagle medium high glucose (DMEM, Biowest) supplemented with 10% FBS, 1.3% L-glutamine and 1.3% Pen/Strep and incubated at 37 °C and 5% CO_2_. For transient transfections, 0.8 × 10^6^ HEK293T cells were seeded in 6-well plates and cultured for 24 h. Cells were transiently transfected with polyethyleneimine (PEI) (Sigma-Aldrich, St. Louis, MO, USA) using a DNA/PEI ratio of 1:4. After 6 h of incubation the transfection medium was replaced with fresh media with or without 30 µM PTC124. To support cilia generation, the medium was replaced 24 h after transfection with FBS-free DMEM with or without 30 µM PTC124 for 48 h. After 48 h the starved cells were used for protein isolation.

### 4.5. Ciliary Staining and Microscopy

For immunostaining, either an uncoated coverslip (fibroblast cells) or a poly-L-lysine (PLL)-coated coverslip (HEK293T cells) was placed at the bottom of a 12 well plate before cell seeding. Cells were fixed in 4% paraformaldehyde for 30 min, washed (3 × 3 min in PBS containing 0.05% Tween-20 (PBS-T)) and incubated for 30 min at 80 °C in 0.1 M Tris-HCL (pH 9) to expose the epitopes of interest. After washing, unspecific antibody binding was blocked by a 30 min incubation in PBS-T containing 2% bovine serum albumin. The primary antibodies were incubated over-night at 4 °C. Before applying the secondary antibodies, conjugated either to Alexa fluor 488 or Alexa fluor 568 (Life Technologies, Carlsbad, CA, USA), the cells were washed with PBS-T. The cells were mounted with Fluoromount containing 4′,6-diamidino-2-phenylindole (DAPI) (SouthernBiotech, Birmingham, AL, USA). The axonemes of primary cilia were stained using rabbit polyclonal anti-detyrosinated α-tubulin antibodies (1:1000; Ab3201, Abcam, Cambridge, UK). RPGR was detected with the rabbit polyclonal RPGR antibody (1:200; HPA001593, Sigma-Aldrich). Images were obtained with a DM6 microscope (Leica, Heerbrugg, Switzerland) using a 63×/1.40 plan apochromat objective and an Orca flash 16-bit camera (Hamamatsu photonics, Hamamatsu City, Japan) at a resolution of 2048 × 2048 pixels. Images were processed using LAS-X (Leica) software and Fiji-ImageJ (https://fiji.sc/).

### 4.6. RNA Isolation and RT-PCR

Total RNA was extracted and isolated from cultured fibroblasts using NucleoSpin RNA kit (Macherey-Nagel, Düren, Germany) according to the manufacturer’s instruction. Using 500 ng total RNA, cDNA was generated by applying random primers and SuperScript III Reverse Transcriptase (Invitrogen). RT-PCRs were performed using HotFire Tag Polymerase (Solis Biodyne, Tartu, Estonia) to amplify a fragment from exon 9 (5′-TCGCCACGGAAAATTAGGACTTG-3′) to 11 (5′-TCTGGCTGCATGAGGTCCTGTTC-3′). PCR products were analyzed on a 2% agarose gel and verified by Sanger sequencing.

### 4.7. Protein Isolation and Western Blotting

Cells were lysed in RIPA buffer (1× PBS, 1% NP40, 0.5% sodiumdeoxycholate, 0.5% SDS) containing phosphatase inhibitor cocktail 2 and 3 (1:100; Sigma-Aldrich) and complete Protease inhibitor cocktail (1:25; Roche, Basel, Switzerland). The cell suspension was incubated for 30 min on ice and centrifuged at 15,000× *g* for 30 min. The protein concentration was measured with the BCA Protein assay Kit (Thermo Fisher Scientific) according to the manufacturer’s instruction. Before separating on a 10% SDS-polyacrylamide gel, the protein lysates were incubated for 5 min at 95 °C in Laemmli buffer. The separated proteins were transferred to a Polyvinylidene difluoride (PVDF)-membrane (0.45 µm pore size; Millipore, Burlington, NJ, USA) by wet blotting at 45 V for 2 h. The membrane was blocked in 5% bovine serum albumin in TBS-T for 1 h and incubated with primary antibodies over night at 4 °C. A secondary horseradish peroxidase-conjugated antibody was used for detection by enhanced chemiluminescence. Proteins could be visualized with the ChemiDoc MP chemiluminescence detection system (BioRad Laboratories, Munich, Germany). RPGR was detected with the rabbit polyclonal RPGR antibody (1:500; HPA001593, Sigma-Aldrich) and the housekeeping gene GAPDH with the mouse monoclonal GAPDH antibody (1:1000; 0508007983, Chemicon International, Temecula, CA, USA).

### 4.8. Statistical Analysis

For quantification of RPGR’s subcellular localization and read-through efficiency, immunofluorescence-based stainings of the primary cilium were analyzed. At least four independent experiments were performed. Random pictures were taken under the microscope and 50 to 100 cells were investigated per experiment. Statistical analyses were performed applying the student’s *t*-test to determine if the groups were significantly different from each other. For Western blot analyses, the quantification of images was done using the software ImageLab 5.2 (BioRad Laboratories). Statistical analysis of band intensities in Western blots was performed using the Mann-Whitney U test. A *p*-value of <0.05 was considered statistically significant.

## Supplementary Materials

Supplementary materials are available online at https://www.mdpi.com/1422-0067/21/22/8418/s1.
