# Supplementary material for: Translational Read-Through Therapy of RPGR Nonsense Mutations
Source: Int J Mol Sci. 2020 Nov 10;21(22):8418. doi: 10.3390/ijms21228418 (PMC7697989; doi:10.3390/ijms21228418)
Supplement: Supplementary file 1 [file ijms-21-08418-s001.pdf]

Supplementary material

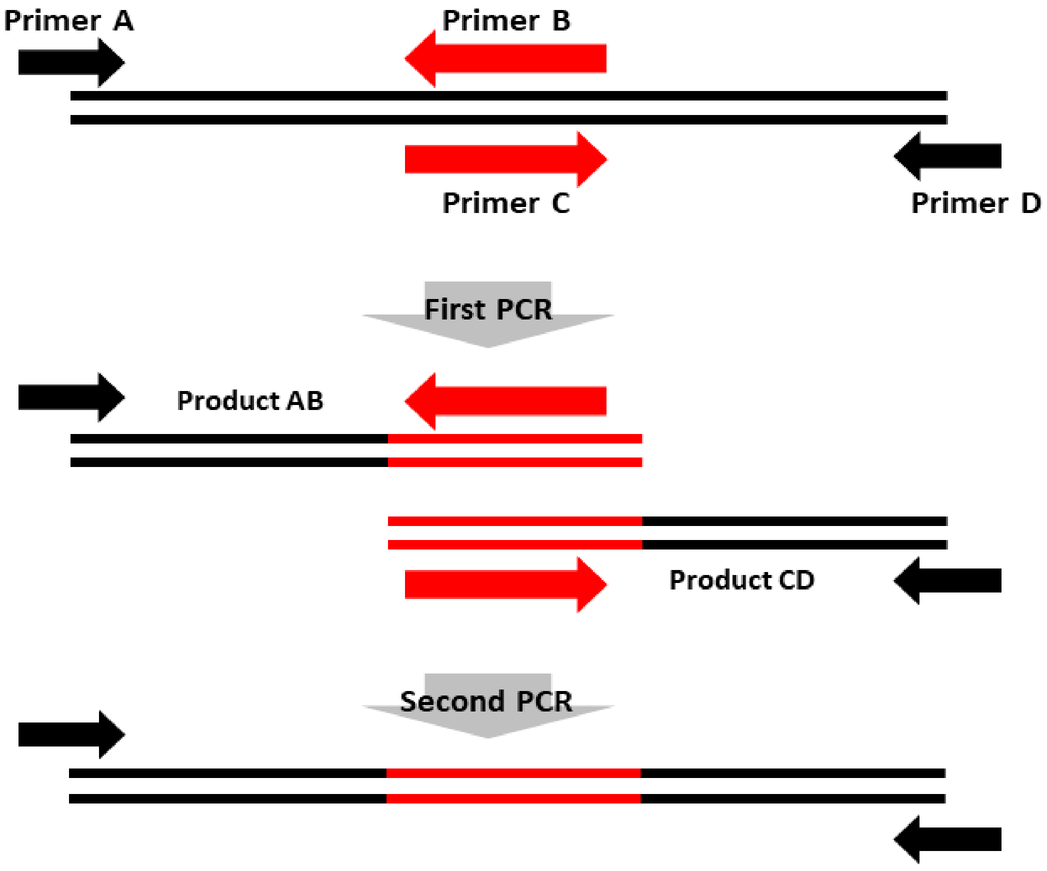

	Primer ID	Sequence 5‘-3‘
Gateway cloning - forward primer	Primer A	GGGGACAAGTTTGTACAAAAAAGCRGGCTTCatgag ggagccggaagag
Gateway cloning - reverse primer	Primer D	GGGGACCACTTTGTACAAGAAAGCTGGGTttatagtat tgtacaggattttgatc
UAA (AS385)	Primer B - UAA	cgatgaaataaatgatacttgctaatctgtggcgacttttctgccg
UAA (AS385)	Primer C - UAA	cggcagaaaagtcgccacagattagcaagtatcatttatttcatcg
UGA (AS385)	Primer B – UGA	cgatgaaataaatgatacttgctgatctgtggcgacttttctgccg
UGA (AS385)	Primer C - UGA	cggcagaaaagtcgccacagatcagcaagtatcatttatttcatcg

Supplementary Figure S1: Site-directed mutagenesis. Generation of RPGR expression constructs encoding the full-length RPGR protein from exon 1 to 19 either with a UAA stop codon at amino acid position 384 or a UGA stop codon at amino acid position 384. The PCR primers B and C contain the mis-matched sequence to insert either a UAA or UGAs top codon in exon 10 of the *RPGR* gene. During the first PCR, the primer combinations A-B and C-D amplified two products with the mutated sequence (red). During the second PCR, the PCR-products AB and CD were used as templates and were amplified with the primer combination A-D. RPGR reference sequence: NM_000328.3

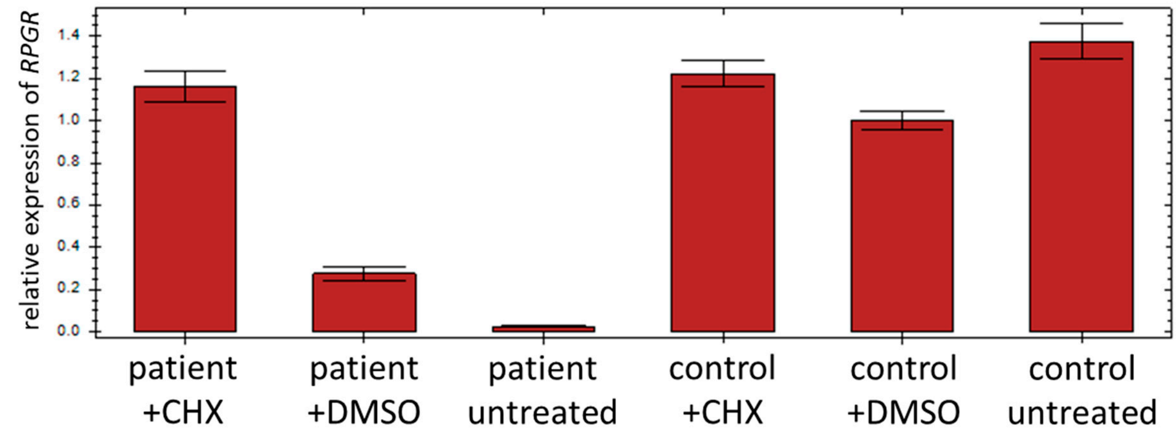

Suppl. figure S2: Quantitative RT-PCR of the cycloheximide treated patient and control cells. Conventional RT-PCR analyses suggested that the *RPGR* transcripts carrying the nonsense mutation c.1154T>A (p.Leu385stop) undergo nonsense-mediated decay (NMD). To inhibit NMD, we applied cycloheximide (CHX, dissolved in DMSO) to both, patient-derived fibroblasts and controls. The quantification was performed using CYBR Green detection of RT-PCR products generated with primers amplifying exons 9 through 11 of *RPGR* (previously published in [1, 2]). *RPGR* expression levels in control fibroblasts were almost independent from the CHX treatment. In contrast, the patient-derived fibroblasts showed strongly increased levels of *RPGR* transcripts due to the CHX treatment. DMSO seems to have smaller effects on *RPGR* expression levels. The quantification was performed relative to *GAPDH* expression levels. The error bars show the standard deviation (3 replicates).

1. Da Costa, R.; Glaus, E.; Tiwari, A.; Kloeckener-Gruissem, B.; Berger, W.; Neidhardt, J., Localizing the RPGR protein along the cilium: a new method to determine efficacies to treat RPGR mutations. *Gene Ther* **2015,** 22, (5), 413-20.
2. Glaus, E.; Schmid, F.; Da Costa, R.; Berger, W.; Neidhardt, J., Gene therapeutic approach using mutation-adapted U1 snRNA to correct a RPGR splice defect in patient-derived cells. *Mol Ther* **2011,** 19, (5), 936-41.
